# A Community Resource Referral Intervention for Informal Caregivers of People With Dementia

**DOI:** 10.1093/geroni/igaa057.485

**Published:** 2020-12-16

**Authors:** Kelly Boyd, Kate Doyle, Soo Borson, Elbert Huang, Stacy Lindau, Aviva Nathan, Katherine Thompson, Jennifer Makelarski

**Affiliations:** 1 The University of Chicago, Chicago, Illinois, United States; 2 University of Chicago, Chicago, Illinois, United States; 3 University of Washing, Seattle, Washington, United States; 4 University of Chicago, Chicago, United States; 5 University of Chicago, Chicag, Illinois, United States; 6 University of Chicago Medicine, Chicago, Illinois, United States

## Abstract

The National Alzheimer’s Project Act (PL-111-375) calls for connecting dementia caregivers to community resources, but few caregiver interventions have focused on this component of support. CRx-D is a theory-driven, scalable intervention that addresses common caregiver needs through: (1) education to normalize needs, (2) coaching on resource activation and (3) tools to promote access, including a printed list of nearby resources (“HealtheRx-D”), an online resource finder, proactive and ongoing text message support. We pretested the intervention arm of CRx-D among 10 caregivers of PWD to assess appropriateness, feasibility and fidelity. Patients presenting for care at 3 outpatient clinics were screened for eligibility, enrolled and given the intervention (12/19-01/20). Of 1,038 patients approached, 12 were caregivers of PWD; 10/12 met all eligibility criteria. All 10 eligible caregivers were enrolled in the study. Caregivers were, on average, 49 years old (range, 29-65), primarily female (n=8), Black/African American (n=9), and half were children of PWD (n=5). At baseline, several caregivers indicated unmet caregiving or health needs (caregiving education [n=7], free/affordable meals [n=5] and financial assistance [n=5]) indicating the intervention’s appropriateness. Intervention components were delivered as intended to all caregivers, although one caregiver provided a landline so could not receive texts. A scalable resource referral intervention for caregivers is appropriate. Delivery is feasible with high fidelity. Identifying caregivers at the point of their own healthcare requires high volume screening. These findings will inform a randomized controlled trial of 344 caregivers of PWD to evaluate the impact of CRx-D on caregiver self-efficacy and satisfaction with care.

